# The Person-Reported Outcome of Conversational Success (PROCS): Tool development and psychometric validation

**DOI:** 10.3758/s13428-026-03063-4

**Published:** 2026-06-10

**Authors:** Camille J. Wynn, Samantha Budge, Carolyn R. Baylor, Tyson S. Barrett, Stephanie A. Borrie

**Affiliations:** 1 Department of Speech and Hearing Sciences, Utah State University, Logan, UT USA; 2 Department of Rehabilitation Medicine, University of Washington, Seattle, WA USA

**Keywords:** Conversation, Communication, Interaction, Social communication

## Abstract

Despite the centrality of successful conversation to well-being, psychometrically developed and validated tools to measure this construct are lacking. The purpose of this study was to develop and validate the Person-Reported Outcome of Conversational Success (PROCS), a measure of conversational success. We define success as the degree to which an interlocutor evaluates a conversation as beneficial, worthwhile, and/or satisfactory. In phase one of development, an initial draft of the PROCS was created based on a literature review, expert input, and established frameworks in communication research. In phase two, cognitive interviews were conducted with 39 participants to assess the measure’s content, format, and usability and further refine the measure. In phase three, 50 participants completed the PROCS in live embodied conversations to assess the measure’s practical effectiveness and usability. Finally, in phase four, psychometric validation was completed using data from 817 participants who completed the PROCS based on recently recalled conversations. The final version of the PROCS can be used to quantify the success of a single conversation. Careful planning of the initial draft of the PROCS coupled with input from participants from a diverse set of user groups across multiple phases of development, ensures the measure’s validity, clarity, usability, and versatility. The PROCS is a valuable tool for assessing conversational success, with applications in both research and clinical practice.

**Supplementary Information:**

The online version contains supplementary material available at 10.3758/s13428-026-03063-4.

## Introduction

Conversation is a fundamental part of life. Through conversation, we build relationships, share stories, exchange ideas, solve problems, and express emotion. Individuals begin engaging in conversational exchanges early in childhood, and conversation continues to play an important role throughout the lifespan. That conversation plays such a central role in our lives is evidenced by the abundance of research demonstrating that the quality and quantity of a person’s social interactions are one of the strongest predictors of their overall well-being (Diener & Seligman, 2002; Epley & Schroeder, 2014; Mehl et al., 2010; Ng et al., 2022; Sun et al., 2020). When people have conversations that they judge as positive, open, and supportive, they report stronger feelings of belonging and unity (Abu Bakar & Sheer, 2013; Koudenburg et al., 2013), higher levels of cooperation (Abu Bakar & Connaughton, 2019), greater emotional improvement (Priem & Solomon, 2015), and more positive relationship outcomes (Brunell et al., 2007).

Given the importance of conversation, it is not surprising that there is a large body of research focused on how to have successful conversations. This research spans many disciplines, including psychology, neuroscience, communications, business, computer science, and speech-language pathology. Disciplines often approach conversation research from different perspectives and draw on various methodological procedures to address questions. Currently, conversational success is defined in various ways across the literature, leading to a range of measurement methodologies (e.g., self-reported ratings; external observer ratings; objective measures of task success, conversational engagement, or message comprehension). Deciding which approach to use is not a straightforward decision. Like many important psychosocial concepts (e.g., happiness, job satisfaction), conversational success represents a latent construct, meaning direct observation or measurement is difficult or impossible. Beneficially, however, psychometric theories (e.g., classical test theory) provide methods to assess the ability of a measure to precisely and meaningfully represent the latent construct of interest. As such, the effectiveness and efficacy of a measure of conversational success can be inferred based on the strength of its psychometric properties.

First, a measure of conversational success must have good construct validity. That is, it should actually measure the underlying theoretical construct of conversational success. Here, we operationally define conversational success as the degree to which an interlocutor evaluates a conversation as beneficial, worthwhile, and/or satisfactory. The evaluation of success may reflect both perceived outcomes (e.g., gaining or sharing desired information) and subjective experience (e.g., a sense of connection with one’s conversational partner; Guydish & Fox Tree, 2021). This definition prioritizes lived experience, emphasizing that conversational success is grounded in an interlocutor’s perceptions of and experiences within a conversation, rather than any external standards or criteria. While existing measures of conversational success exist, they are often not based on the interlocutor’s lived experience. For instance, some studies rely on objective measures of external behaviors that are intended to act as proxies for conversational success. Researchers have measured the length of pauses between speaking turns, the number of interruptions, or the frequency of conversational repair strategies (e.g., Levitan et al., 2012; Tetnowski et al., 2021). While these behaviors may be indicative of some aspect of success (as defined by personal experience) in some contexts, this is not always the case. For example, Templeton and colleagues (2023) found that while long pauses between speaker turns signaled discomfort and awkwardness in conversations between strangers, they were a sign of reflection and connection in conversations between friends. Conversations are also frequently evaluated using subjective measures such as surveys and rating scales. Many of these measures rely on ratings made by outside observers who assess the conversation based on a transcript, audio recording, or video recording (e.g., Guydish & Fox Tree et al., 2022a; Wynn et al., 2022; Borrie et al., 2019; Spitzberg & Adams, 1995). However, the external behaviors observed by outside observers may not accurately reflect the experiences and internal perceptions of the conversational participants. Indeed, ratings made by outside observers of components of conversational success, such as conversational involvement and depth, are only weakly related to the ratings of these same constructs made by interlocutors within the conversation (Priem & Solomon, 2018; Sun et al., 2020).

Existing measures of conversational success also include those that rely on self-report—following a conversation, interlocutors respond to questions about their interaction (e.g., Speer et al., 2024; See et al., 2019). Such measures (often referred to as person-reported outcome measures [PROMS]) are advantageous in that they provide a more valid representation of the personal experiences of the people in these conversations (Yorkston & Baylor, 2019). However, most existing person-reported measures of conversational success have not undergone any rigorous development or evaluation. The key here is that an extensive literature review, an expert panel, cognitive interviewing, and pilot testing are needed to ensure that tool items capture the aspects of conversation that people feel are most important to a successful conversation. Additionally, potential issues of bias (i.e., items produce differential responses based on characteristics of the responder that should not impact conversational success) and ambiguous or unclear item wording may exist (Speer et al., 2024; See et al., 2019).

A rigorous measure of conversational success must also have good content validity. That is, the measure must be relevant and representative of all the facets of conversational success. Again, many current approaches fall short in this regard. For instance, studies (including our own previous research) have attempted to capture success by quantifying the efficiency with which interlocutors complete a given conversational task (e.g., Borrie et al., 2019; Pardo et al., 2019), or the accuracy with which information is transferred between partners (Crompton et al., 2020; Oates et al., 2024). These types of measures do capture concepts that are presumably related to success (i.e., accurate and timely exchange of knowledge and collaborative problem-solving) and provide useful information regarding these conversational attributes. However, the scope of these measures is relatively narrow and thus unlikely to capture the entirety of the construct of conversational success. Other approaches that have used subjective rating scales often only include one or a couple of items (e.g., Borrie & Delfino, 2017; Wynn et al., 2023), again, limiting the extent to which a multifaceted construct can be captured. As highlighted above, even those measures that do use multiple items rarely rely on any type of systematic validation process (extensive literature review, expert panel, cognitive interviewing, pilot testing) to ensure that the full breadth of the construct is adequately represented.

Measures of conversational success should also be ecologically valid. That is, the measure should be broadly applicable to the types and variety of conversations that are relevant and meaningful to people in their everyday lives. Additionally, this type of measure will be most effective if it is useful and versatile across a range of conversational contexts. Currently, many conversational measures were designed for the performance of a certain task and thus are only valid for that specific task (e.g., Map Task [Anderson et al., 1984; Anderson et al., 1991] Alien Game [Tylén et al., 2023]). Many self-reported measures are similarly only applicable to a certain type of conversation (e.g., questions about laughing easily that would not apply to serious conversations [Hecht, 1978]). As such, these types of measures cannot be used to study success in different conversational settings and/or directly compare success across contexts, and they may entirely miss the conversational contexts that are most important in people’s lives.

In addition to versatility in conversational settings, measures of conversational success are optimally inclusive when they include items that are broadly applicable to many different populations. Thus, measure development should include the thoughts and ideas of people with a wide variety of conversational experiences—people for whom high-quality conversations occur frequently and conversational success is easily achieved, and also those for whom conversation is more difficult and effortful. One group of people whose perceptions are especially important is those with disorders or disabilities that make communication more challenging. People with a wide variety of communication disorders (e.g., dysarthria, voice disorders, hearing loss, aphasia) often report having conversations that are less effective, meaningful, and/or fulfilling (e.g., Baylor et al., 2011; Johansson et al., 2011; Saldert & Bauer, 2017). Thus, considering the experiences and ideas of people within these populations when developing a person-reported measure of conversational success will increase the relevance and inclusivity of this type of tool and the degree to which it can be used to understand the conversations of a wide variety of people, both with and without communication disorders. Lastly, measures that can be used to assess conversational success in clinical evaluation and treatment settings are still lacking. Thus, considering the perspectives of individuals with communication disorders in measure development also increases the clinical utility of the measure, providing clinicians with a tool that can be used for evaluation, treatment, and progress monitoring in conversational contexts.

In addition to these aspects of tool development, other psychometric properties must be verified through quantitative evaluation. Developers should ensure that the measure is reliable and demonstrates strong convergent validity (i.e., it is correlated with other constructs with which it theoretically should be) and discriminant validity (i.e., it is unrelated to constructs from which it should theoretically be independent). Further, the structure of the measurement tool (e.g., number of factors, item factor loadings, issues of item variability, correlated errors) should be established. These inform whether all items are relevant and necessary and whether all items represent a single construct or if there are sub-groupings of items representing different constructs. However, current tools for measuring conversational success rarely undergo this type of evaluation. Without an understanding of these features of a measure, we are at risk of making conclusions that are prone to a series of issues. For instance, without estimates of reliability for a measure, random measurement error can reduce statistical power (Blake & Gangestad, 2020; Kanyongo et al., 2007) and can attenuate or exaggerate effect size estimates (Hyslop & Imbens, 2001) unbeknownst to the researcher.

### Present study

As with any latent construct, measurement of conversational success demands careful and systematic development and must undergo rigorous psychometric validation to ensure its validity and reliability. Yet, in our review of the literature, we found almost no measures that met these requirements. Indeed, most measures currently in use have not undergone psychometric validation. This significantly reduces the degree to which these measures can be considered to provide accurate, comprehensive, and reliable information regarding conversational success. Without such a measure, existing research efforts to understand conversational success may be somewhat premature. Accordingly, the purpose of this project was to develop and validate a tool to measure conversational success. This tool, the *Person-Reported Outcome of Conversational Success (PROCS),* was developed and validated through a systematic multi-step process that includes four critical phases: 1) initial draft tool development, 2) cognitive interviews, 3) tool assessment in embodied conversations, and 4) psychometric validation. The result is a tool that can be used for both research and clinical purposes to understand the experiences of individuals (both neurotypical individuals and those with disorders or disabilities that impact communication) during embodied conversations.

## Methods and results

The methods and results of this manuscript are organized into four sections corresponding to the four phases of tool development and validation introduced above. The methods and results for each study phase are presented together. The development of this tool is based on commonly accepted best practices in measure development (e.g., Patient-Reported Outcome Measurement Information System (PROMIS) Health Organization, 2013). Processes, which were often iterative with ongoing refinement, included defining the target concept (phases one and two), individual item composition (phases one, two, and three), item pool construction (phases one, two, and three), instrument formatting (phases one, two, and three), determination of item bank properties (phase four), analysis of validity (phase four), and analysis of reliability (phase four).

### Phase one: Initial draft tool development

Phase one was focused on the initial development of the PROCS. In line with the project’s overall purpose, we determined that the project should be organized so that the respondent evaluates a specific conversation in which they participated. To do this, they rate the conversation on a number of indicators of conversational success. With this in mind, development of the PROCS first required considering the content of the item bank (i.e., items that best reflect success and should thus be rated by respondents), and the overall structure (i.e., stem phrasing, response format, scoring method) of the measure.

#### Methods

To develop candidate items for the item bank, key dimensions of conversational success were first identified from established tools (e.g., Eadie et al., 2006; Baylor et al., 2013; Bienvenu, 1971; Sedikides et al., 1999; Baylor et al., 2024; Rosen et al., 2004), published qualitative interviews (Baylor et al., 2011; Davidson et al., 2008; Howe et al., 2008; Yorkston et al., 2007, 2008), and discussions among the research team members, all of whom are clinicians and/or researchers with expertise in conversation and social interaction. Following this process, we created a list of candidate items. In doing so, we aimed to select enough items to cover the breadth of the construct of conversational success while keeping the measure parsimonious and avoiding redundancy. Care was also taken to ensure that items were applicable across a range of conversational contexts (e.g., items about getting to know one’s conversational partner were not included as these items are not applicable to partners who already know each other). We also aimed to phrase items clearly, without ambiguity, neutrally (i.e., not leading respondents to answer in any particular manner), and in a way that was free from bias. In addition to item generation, we also considered the way in which these candidate items would be presented and scored.

#### Results

We generated two initial draft versions of the PROCS. Each version contained the same 14 candidate items. However, they differed in the stem phrasing used to introduce each candidate item. One version (termed the *Difficulty* version) relied upon the phrasing used in previously established and validated measures (i.e., updated versions of the Communicative Participation Item Bank [CPIB]; Baylor et al., 2024). Specific phrasing for this version was “How difficult was it for you to…”. The second version (termed the *Ability* version) was motivated by item development guidelines, which suggest using positively worded stem phrases (Ford & Scandura, 2018). Specific phrasing for this version was “I was able to…”. In line with the response format of the CPIB, participants rated each candidate item on a four-point scale. However, the response option wording varied depending on the phrase used (*Difficulty* version: “Not Difficult at All” to “Very Difficult”; *Ability* version: “Strongly Disagree” to “Strongly Agree”). For each draft, candidate items were each scored according to rank order on a scale from 0–3 with 0 indicating low success and 3 indicating high success. Scores from each candidate item were then summed, yielding a total score between 0 (very unsuccessful) and 42 (very successful).

### Phase two: Cognitive interviews

Phase two consisted of cognitive interviews, which were conducted to seek feedback from people representing potential user groups regarding the content, format, and usability of the measure. The cognitive interview process is in line with best-practice guidelines for tool development (Patient-Reported Outcome Measurement Information System (PROMIS) Health Organization, 2013) and was directly influenced by established qualitative interview studies and previously published guidelines and techniques (Baylor et al., 2011; Davidson et al., 2008; Howe et al., 2008; Willis, 2005; Yorkston et al., 2007, 2008). Data for this phase of the study were collected with ethical approval from the institutional review board at Utah State University (protocol # 12,655). Written informed consent was obtained from all participants.

#### Methods

##### Participants

Data for this phase were collected from a total of 39 participants (13 men, 25 women, one non-binary person) between the ages of 23 and 84 years (*M* = 63.0, *SD* = 18.4). These participants represented a diverse group including 15 people with various communication disorders (i.e., hearing loss, dysarthria, voice disorder, mild aphasia, mild cognitive communication disorder), 12 certified speech-language pathologists^1^ who work in a variety of settings (e.g., private practice, home health, acute care, skilled nursing, university clinics, and public schools), and 13 individuals with no history of a communication disorder and no clinical background in speech-language pathology. More information about participants can be found in the supplementary material. To gather feedback from people with diverse life experiences and perspectives, participants were recruited from many sources, including university clinics, local support groups, online forums, participant databases, and word of mouth. Thus, participants varied considerably across many different demographic factors. Upon completing the study, all participants received a gift card for their participation.

##### Procedure

Cognitive interviews were conducted with individual participants in a face-to-face setting and were recorded using Zoom H4N recorders. All interviews were conducted by the second author (Budge), with a second research assistant present for notetaking and providing interview assistance. Each interview followed a basic structure; however, participants were encouraged to think out loud throughout the interview, which often led to new avenues of discussion, resulting in some variation among interviews.

To assess the content validity of the PROCS (i.e., to ensure it measured what participants perceived as important in a conversation), participants were first asked what success in a conversation looked or felt like. If participants needed more prompting or indicated they did not understand the question, they were asked to reflect on successful conversations they had participated in and describe those interactions. These questions were presented before the participants were shown the draft of the PROCS to ensure the ideas presented were candid and not biased by familiarity with the measure.

After defining conversational success, each participant was prompted to think about a recent conversation that they had in which they could clearly remember the details and how they felt about the interaction. Participants were told that the conversations they chose should be one-on-one and in a face-to-face setting. However, no other criteria were given. After selecting a conversation, participants were asked to complete one of the two versions (i.e., *Difficulty* version or *Ability* version) of the PROCS. In order to get a valid observation of how participants responded to items, the interviewer did not provide any further guidance in filling out the measure. After this was complete, participants were asked about their first impressions of the items. Prompts included asking participants whether aspects of the measure “stuck out” to them (in either a positive or negative way), whether questions were difficult to understand, and whether any question was particularly hard to answer. Participants were also asked about the format in which the items were presented, including the stem phrasing used to introduce each item and the response scale used to rate each item.

Following an in-depth discussion about the PROCS draft they had initially used, the participants then completed the other version for the same conversation (i.e., participants who originally filled out the *Difficulty* version then filled out the *Ability* version). The order of the draft versions presented was counterbalanced across participants to account for potential order effects. Once complete, participants were asked about their thoughts on the different stem phrases used and which version of the tool they preferred. The preferred version was scored, and participants were asked if their score “made sense” or “felt right” in terms of representing the success of that conversation.

Participants were then asked to think of another recent conversation. However, this time they were asked to select a conversation with a different outcome (e.g., if participants had initially discussed a positive interaction, they were prompted to think about a conversation that had gone poorly, or vice versa). Participants completed the measure again using their preferred version and were asked for comments and feedback. As before, the PROCS was scored, and participants were again asked if their score aligned with their perceptions of the conversation.

The interviews concluded by revisiting notes from earlier in the interviews when participants had been asked about how they judged conversational success before they had seen the drafts of the PROCS. Any concepts mentioned by participants in that initial conversation that had not been explicitly included in the candidate items were revisited, and participants were asked whether those ideas had been appropriately addressed in the measure. Participants were invited and encouraged to submit candidate items for consideration. Finally, all participants were asked to “imagine that [they] were making [their] own version of this measure” and to indicate which candidate items they would keep and which they would remove (if any).

##### Data analysis

All 39 interviews were transcribed using an online transcription service (Trint, 2022). Each conversation was then fully reviewed by a research assistant who corrected any inaccuracies. Following transcription, comments from the initial discussion about conversational success (before participants saw the measure) were reviewed and discussed by the research team to ensure that participants’ thoughts and ideas about conversational success were adequately represented within the PROCS. Additionally, individual participants’ feedback regarding the measure was entered into a spreadsheet, indicating either no concerns or recommended changes for each item. In addition to the feedback regarding item content, comments that addressed the format of the PROCS were also noted. Following this careful review of participant feedback and extensive discussion among the research team, changes were made to the initial drafts.

Several steps were taken to promote trustworthiness and credibility of the data. First, all interviews were facilitated by the second author to provide consistency across interviews. Second, each individual transcript was reviewed by research assistants for accuracy. Next, the research team debriefed regularly to review key findings and to determine if any new questions should be raised in later groups based on emerging findings. Finally, the research team employed triangulation by including multiple researchers with diverse clinical and research backgrounds in the analysis.

#### Results

##### Candidate items

Participants offered many important insights that led to several changes to the initial version of the PROCS (summarized in Table 1). Here, we note that, in most cases, the ideas expressed by participants did not systematically differ across participant group, indicating that many components of conversational success are widely applicable and generalizable. First, multiple participants voiced concerns about several candidate items that felt redundant, overly forceful, or irrelevant to their lived experience of conversational success. Further, many SLP participants noted a desire to have a tool that was brief and concise, increasing its adoption in clinical practice. Accordingly, a total of five items were removed from the measure (see Table 1). In addition, participants expressed concerns that some items were not appropriate for all contexts. For instance, one participant noted that it felt inappropriate to ask questions or provide information while listening to her coworker express frustration about their manager, and that doing so would have fostered an unsuccessful rather than successful conversation. Another participant suggested that in some instances, such as speaking with a stranger at the grocery store, engaging in small talk would be more appropriate and successful than trying to foster a feeling of deep connection. Accordingly, four items were reworded to include the phrase “*as desired”.* Finally, some participants suggested adding an item that directly asked about the degree to which they felt they were able to *participate* in the conversation. The theme of participation (e.g., adequate turn-taking, one person not dominating the conversation) also came up frequently in interview questions about what made a conversation successful (see Guydish & Fox Tree, 2022b; Guydish et al., 2021 for relevant observations in the literature). As such, an item regarding participation was added to the measure.

**Table 1:** Changes made to candidate items after phase two

Candidate item [Reference Phrase]	Change made	Justification	Example quotes
“…engage at a level [I] felt good about” [Engage]	No change made	Item aligned with participants’ perceptions of success	“It varies [between conversations] how engaged I’m able to be.”—P01 “One [person] can just be listening as long as they’re really engaged. They both have to be engaged. You need to be present.”—P10
“…keep up with the conversation” [Keep up]	No change made	Item aligned with participants’ perceptions of success	“Without my hearing aids, [keeping up with the conversation] would’ve been difficult depending on the surroundings.”—P31 “My big fear is that I’m not going to be able to [keep up]. I don’t ever want people to think, ‘well, she can’t keep up’. But I lose words too, so that makes it hard.”—P34
“…feel heard by [my] partner” [Feel heard]	No change made	Item aligned with participants’ perceptions of success	“The person I’m speaking to is listening and understanding what I’m saying—not thinking of what their response is going to be.”—P21 “Did I feel heard? Oh, that’s a big one. The kind of feelings that come with conversations really affects your outcomes.”—P27
“…present [myself] the way [I] wanted to” [Present]	No change made	Item aligned with participants’ perceptions of success	“Present yourself the way you wanted to... for the gender diverse or the LGBT community that’s really important. I honestly want to say that that’s somewhat difficult for me. I think I still struggle with that.”—P12
“…say what [I] wanted to say” [Say]	No change made	Item aligned with participants’ perceptions of success	“A lot of times I just want to swear up a storm and just be mad. But I’m not going to because that might not be received well.”—P27
“…participate” [Participate]	Added	Requested by participants	“[Success happens when] I can participate and provide feedback”—P039 “If both people aren’t interacting, is it really a conversation?”—P10
“…ask questions” [Questions]	Changed to “…ask questions as desired”	Not applicable in all situations	“Asking questions may or may not have been applicable at times during the conversation.”—P26 “As [Parkinson’s disease] goes on, it becomes more difficult to vocalize your questions. And so at a certain point I probably will have difficulty in asking questions.”—P32
“…share [my] opinion” [Opinion]	Changed to “…share [my] opinion as desired”	Not applicable in all situations	“It’s not always appropriate for me to share my opinion or make myself heard because my role in some conversations is to be the listener.”—P26
“…share information” [Information]	Changed to “…share information as desired”	Not applicable in all situations	“Letting a coworker vent to you about their day could be a really successful conversation even if you didn’t contribute much.”—P22
“…connect with [my] partner” [Connect]	Changed to “…connect with [my] partner as desired”	Not applicable in all situations	“[A good conversation involves] hearing, responding, making connections, being understood, and staying in a natural flow.”—P37 “We were not only physically distanced, but otherwise distanced. It was kind of like going through the motions, but not ever connecting.”—P21 “I’m here to support you as a person, and you're here to support me. We have that shared value. I feel like our connection grows because we have that ability to be vulnerable together.” -P12
“…make contributions”	Removed item	Redundant with “…ask questions”, “…share [my] opinion”, and “…share information”	“I don’t necessarily dislike [this item], I just think a lot of the other questions play into that.”—P26 “‘Ask questions’, ‘share my opinion’, and ‘share information’ all go back to making contributions.” -P29
“…get information from [my] partner”	Removed item	Redundant with “…share information”	“Maybe you can combine ‘get information’ and ‘give information’ into one question.”—P7
“…give information to [my] partner”	Removed item	Redundant with “…share information”	“You have duplicates with share information versus give information. It’s just another way to ask the same question.”—P32
“…make [myself] heard”	Removed item	Perceived as overly forceful and redundant with “…feel heard by [my] partner”	“This [item] feels a little forceful. It sounds like a bully in a group project.”—P22 “When I first read [the item] I thought, “Did I make, like force myself to be heard?”—P12
“…participate without feeling fatigued”	Removed item	Perceived as irrelevant to conversational success (to both participants with and without communication disorders)	“I can see how somebody could be exhausted with the conversation. I guess it depends on how long the conversation is and how animated it is. Quite honestly, if I get to that point, I’m not having it. I’m not worried about it. I can’t imagine being physically fatigued with any conversation.”—P4 “I don’t remember ever feeling fatigued in a conversation.”—P6

Reference phrase denotes the phrase used throughout the manuscript and in subsequent tables and figures to refer to specific candidate items

Participants (across all groups) stated that all other items were applicable to their lives, easy to understand, and relevant and important components of conversational success. Further, analysis of participants’ descriptions of what conversational success meant to them (prior to seeing the PROCS) indicated that most of the major dimensions of success discussed by participants were directly addressed by one or more of the items. Examples of statements provided by participants that validate the use of specific candidate items can be viewed in Table 1. During the analysis, researchers noted one concept that came up consistently in discussions about conversational success but was not directly addressed in the PROCS. Participants often noted that a conversation was successful when their communication partner faced them, made eye contact, nodded or shook their head when appropriate, and used other physical signals that indicated they were open to that interaction. However, when participants were asked if they thought an item about body language should be added, most responded that they felt that other items (e.g., “...feel heard by [my] partner”, “...connect with [my] partner as desired”) adequately covered this concept and that an additional question was unnecessary. Therefore, no items regarding body language were added.

Beyond gaining feedback from participants, we also wanted to ensure that candidate items adequately captured distinctions between successful and unsuccessful conversations. Recall that participants filled out the same form twice—once for a positive conversation and once for a negative conversation. Accordingly, we ran a linear mixed effects model to see if participant scores varied by type (while accounting for repeated measures across participants). Model results showed a significant difference between scores of positive and negative conversations for both the *Difficulty* (*b* = 15.68, *p* <.001) and *Ability* (*b* = 14.73, *p* <.001) versions of the PROCS, indicating that both versions captured distinctions between successful and unsuccessful conversations.

##### Measure structure

When asked if participants preferred the *Difficulty* version or *Ability* version of the PROCS, no participant expressed strong feelings in favor of one or the other, and many stated that they did not want to choose between the two presented stems. However, some participants with communication disorders expressed appreciation for the *Difficulty* stem because it acknowledges that conversation can be difficult for them. When forced to pick one version over the other, comparable numbers of participants picked the *Difficulty* and A*bility* versions. Further, participants indicated that the stem phrases used to introduce the item did not change their perception of the item or what considerations they made when responding. Spearman’s correlation analysis indicated a significant correlation (*r* =.85, *p* <.001) between participants’ scores on the *Difficulty* or *Ability* version of the PROCS (when filling out each version for the same conversation). Accordingly, given that there was no strong consensus or preference among participants about either version, the research team opted to use the *Difficulty* version as our qualitative interview data supported its acknowledgment of the experiences of participants with communication disorders, and because it is consistent with other tools (i.e., CPIB) that have been shown to be effective in capturing communication metrics.

While there was not a strong consensus regarding stem phrasing, some participants noted that they preferred the “Strongly Disagree” to “Strongly Agree” response format over the “Very Difficult” to “Not Difficult at All” wording because it was more familiar to them. Further, when asked if the scores that they obtained from the measure were representative of how successful their conversations were, all participants stated in the affirmative. However, many participants noted that they wished there were a six-point response option rather than the current four-point options. Accordingly, three new versions of the PROCS were developed. All drafts contained the revised version of 10 candidate items and used stem phrases related to *Difficulty* rather than *Ability*. The first version (termed *Four-point Difficulty* version) kept the original wording of both the stem phrase and response format^2^ as the original *Difficulty* version used in phase two. The second version (termed *Four-point Agreement* version) worded the stem phrase as a statement rather than question (i.e., “It is difficult for me to…”) with a response format on a four-point scale from “Strongly Disagree” to “Strongly Agree”. The third version (*Six-point Agreement* version) used the same statement phrasing, but with a response format on a six-point scale (again from “Strongly Disagree” to “Strongly Agree”). These three versions underwent further evaluation in phase three as described below.

### Phase three: Tool assessment in embodied conversations

The purpose of phase three was to evaluate the practical effectiveness and usability of the PROCS in a naturalistic context where participants evaluate embodied conversation in real time (Patient-Reported Outcome Measurement Information System (PROMIS) Health Organization, 2013). Data collection for this phase of the study was carried out with ethical approval from the institutional review board at Utah State University (protocol # 12,515). Written indication of informed consent was obtained for all participants.

#### Methods

##### Participants

Data for this phase were collected as part of a larger project exploring conversational outcomes across varying contexts using an independent sample from that used in phase two. For this portion of the project, data were collected from 50 participants (20 men, 30 women) between the ages of 30 and 90 years (*M* = 67.9, *SD* = 13.9). This included both people with communication disorders (in this instance, individuals with hypokinetic dysarthria (*n* = 10)) and those without communication disorders (*n* = 40). More information about participants can be found in the supplementary material. Participants were recruited via local support groups, community centers, and relevant online forums, and received a gift card for their involvement in the study.

##### Procedure

In this phase, multiple participants came into the study at the same time. Participants then engaged in multiple embodied dyadic conversations with other participants. Each conversation took place in a private room with only the conversational dyad and a research assistant present. After each conversation, participants evaluated the conversation using the PROCS. To test the measure’s performance in a wide variety of settings, conversations varied in a number of ways. First, the dyadic makeup was not uniform. In some conversations, participants engaged in a conversation with a friend or family member (who was also a participant in the study); in others, they conversed with a stranger (i.e., an unfamiliar communication partner). Dyads also varied in their gender, age, and diagnostic group (i.e., participants with vs. without a communication disorder) composition. Additionally, the type of conversation varied; each participant engaged in two different types of conversations—informational and relational—with each partner. Informational conversations were elicited using the Diapix task (Baker & Hazan, 2011). This is a collaborative spot-the-difference task where conversational partners are given similar images and work together to identify ten differences in those images without showing their partner their picture. Relational conversations were elicited using an adapted version of the Relationship Closeness Induction Task (RCIT) (Sedikides et al., 1999). In this task, participants engage in conversations with each other using a standardized list of questions aimed at helping them learn more about the other person. Both the informational and the relational conversations were 10 min in length, and the order of the two types of conversations was counterbalanced across dyads. Based on scheduling demands and availability, participants conversed with between one and three partners, yielding a total of two to six conversations per participant.

Immediately following each conversation, participants were asked to independently complete one of the three updated versions of the PROCS. As each participant had multiple conversations, all participants completed at least two different versions of the PROCS. Participants who had more than two conversations completed all three versions (sometimes more than once). However, the specific versions each participant completed and the order in which they completed them were randomized. In order to evaluate the measure’s usability, research assistants were instructed not to give any instructions (beyond asking them to fill out the form) to participants. However, they were told to note any points of confusion participants had when filling out the form or any clarifying questions asked by participants. After they had completed each conversation, participants were asked if they had any feedback about the PROCS and if there were any questions they found unclear or difficult to answer. Again, research assistants were instructed to note participant responses.

#### Results

##### Candidate items

A total of 214 measures were completed following real-time conversations. This included 75 of the *Four-point Difficulty* version, 68 of the *Four-point Agreement* version, and 71 of the *Six-point Agreement*. While filling out the form, no participant indicated any misunderstandings regarding any of the items. When asked for feedback, participants stated that candidate items were clear and there were no points of confusion. Accordingly, no candidate items were modified or removed.

##### Measure structure

No negative feedback was given regarding stem phrasing or response scale for any version of the PROCS. Beyond seeking participant feedback, we also looked at each versions’ ability to capture differences across conversations. Because different versions included different scales and different total scores (i.e., total score of 30 for the *Four-point Difficulty* and *Four-point Agreement* version and 50 for the *Six-point Agreement* version), it was difficult to directly compare versions against each other. However, we made a few key observations.

**Fig. 1:**
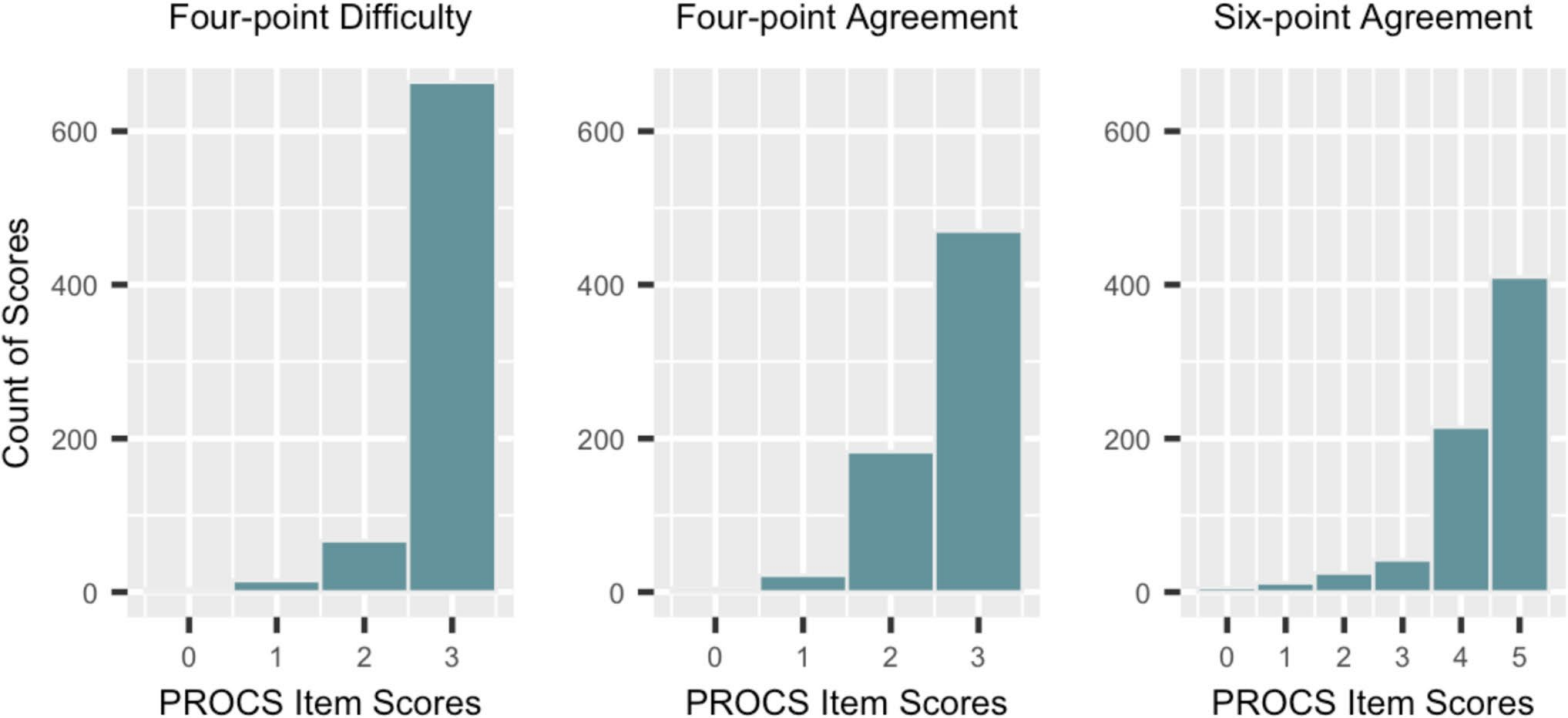
Distribution of individual item responses across all phase three versions of the PROCS

First, we examined differences in the distribution of individual item responses across all three versions of the PROCS. The greater the distribution of responses across the full scale, it is assumed, the greater the ability to capture variability between conversations. Individual item responses across all versions of the measure were negatively skewed, with more items being given high scores (i.e., scores indicative of success) than low scores. However, there was more variability in the distribution of scores for the *Six-point Agreement* version than either of the *Four-point* versions (see Fig. 1). For instance, the percentage of responses at the highest scored category (e.g., “Not difficult at all”/“Strongly disagree”) was lower for the *Six-point Agreement* version (58%) than for either the *Four-point Difficulty* (89%) or *Four-point Agreement* (69%) versions. This suggests the utility of having a Likert scale with a larger range of points.

**Fig. 2:**
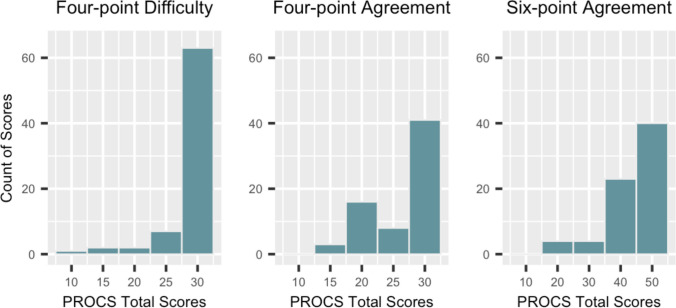
Distribution of total scores across all phase three versions of the PROCS

Additionally, we examined differences in the distribution of total scores across all three versions of the PROCS (see Fig. 2). This analysis showed that the mean score was closer to midline (indicating more varied responses across the full scale) for the *Six-point Agreement* version (*M* = 43.49 out of a possible 50 points [86.99%]) than the *Four-point Difficulty* (*M* = 28.55 out of a possible 30 points [95.15%]) or *Four-point Agreement* (*M* = 26.44 out of a possible 30 points [88.13%]) versions. Further, when standardizing by dividing by the total possible points, the standard deviation was larger for the *Six-point Agreement* version (*SD* = 8.33 out of 50 points [16.7%]) than for the *Four-point Difficulty* (SD = 3.68 out of 30 points [12.3%]) or *Four-point Agreement* (*SD* = 4.67 out of 30 points [15.6%]). Although on their own, these metrics are not conclusive, together they suggest that the *Six-point Agreement* version may capture more information than the others.

**Fig. 3:**
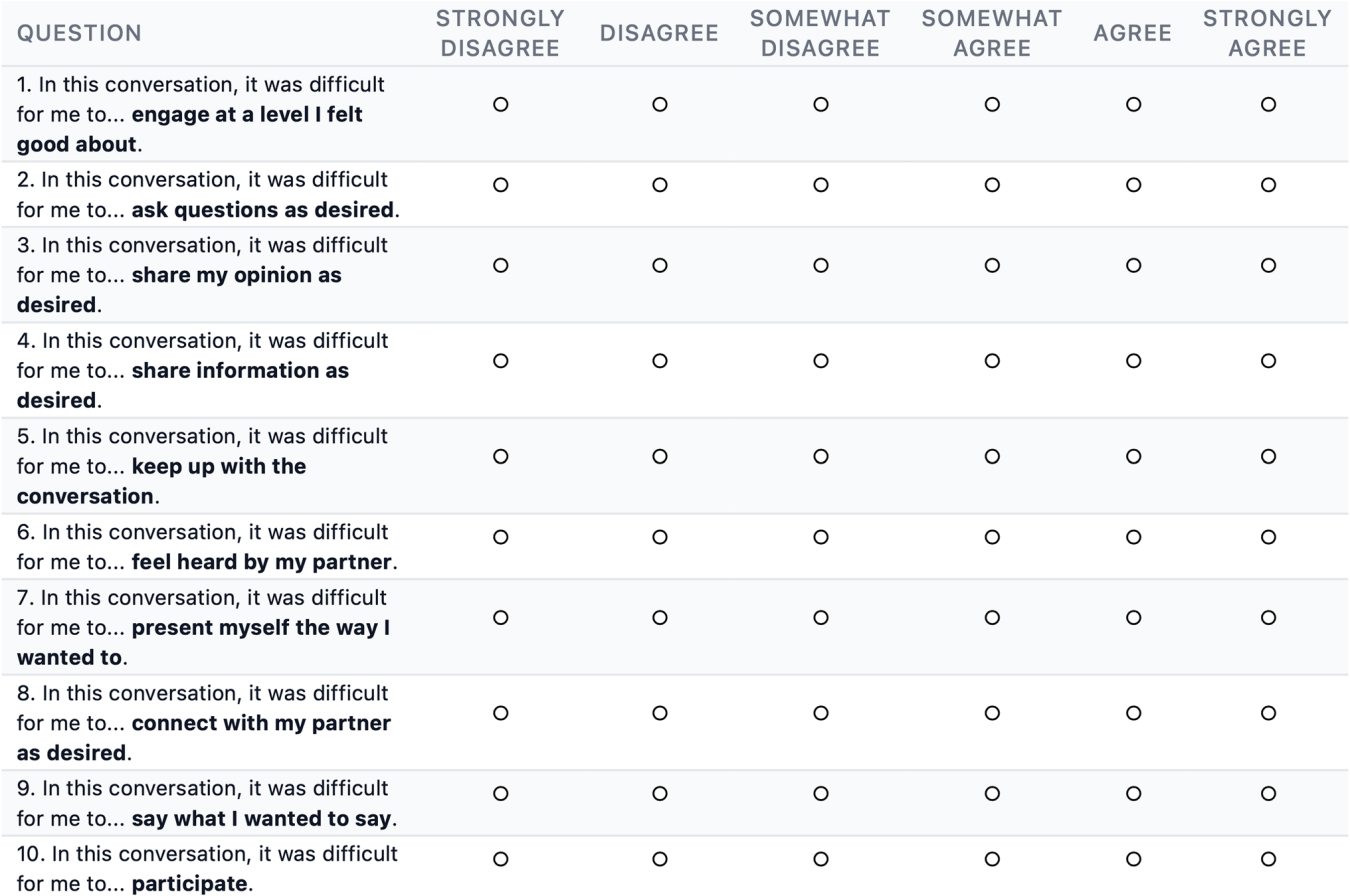
Finalized version of the PROCS used in phase four

It is possible that the observed scores may be impacted by the type of conversation being conducted. In order to examine if this was the case for any of the versions of the PROCS under consideration, we used a linear mixed effects model to see if participant scores varied by conversation type (while accounting for repeated measures across participants). Findings showed no significant difference between scores in the informational and relational conversations for the *six-point agreement* version (*b* = – 1.3, *p* =.42), *four-point difficulty* version (*b* =.26, *p* =.53), and *four-point agreement* version (*b* =.76, *p* =.46). Thus, across all drafts, there was no evidence that conversations varied by conversation type. Accordingly, based on a more even distribution of candidate item ratings, greater variability in total scores, and the preferences expressed by participants in phase two, the *Six-point Agreement version* was selected for the final version of the PROCS (see Fig. 3).^3^

### Phase four: Psychometric validation

The purpose of this final phase was to complete formal psychometric validation of the PROCS. This included determining the structure (e.g., number of factors, loading of items), reliability, convergent validity, and divergent validity of the measure (Patient-Reported Outcome Measurement Information System (PROMIS) Health Organization, 2013). In addition, we assessed whether the measure can be well represented by a summed score. Data collection for this phase was carried out with ethical approval from the institutional review board at Utah State University (protocol # 12,655). Informed consent was obtained for all participants.

#### Methods

##### Participants

Two distinct samples were collected, the first to complete exploratory factor analysis and related assessment of the measure and second to conduct confirmatory factor analysis to validate the structure and performance of the measure. For the first, participants consisted of 409 adults (55.5% men, 43.8% women, less than 1% non-binary) between the ages of 19 and 73, with a mean age of 37. An additional 26 participants were removed because they did not meet the high-quality data standards described below. For the second, participants consisted of 408 adults (53.9% men, 44.9% women, 1.2% non-binary or preferred not to say) between the ages of 19 and 76, with a mean age of 43. An additional 16 participants were removed because they did not meet the high-quality data standards described below. For both samples, all participants were native speakers of American English. While tool development consisted of both people with and without communication disorders, the tool validation (i.e., the current stage) focused specifically on neurotypical individuals with no reported speech, language, cognitive, hearing, or voice impairment. All participants were recruited via Prolific (Prolific, 2023) to encourage a representative sample of the United States. Each participant was compensated through Prolific for involvement in this study.

##### Data collection

All participants in this phase completed a survey created in Qualtrics (Qualtrics, 2023). Participants first answered some basic demographic questions. They were then asked to think about a recent conversation that they could remember well. After providing details regarding the topic, goal, and setting of the conversation, participants were asked three overarching questions (used for subsequent convergent validity analyses). Specifically, participants were asked how successful their conversation was (rated on a Likert scale from 1 to 5), if they considered the conversation to be a good conversation, and if they would like to have a similar conversation again. Following these questions, participants completed the final version of the PROCS (see Fig. 3) about the conversation that they had selected. Several measures were taken to better ensure the collection of high-quality data during this phase. First, Prolific settings were adjusted to ensure research participants were only able to complete the study one time. Next, several attention checks were included that cross-examined information that had been provided in responses to other survey questions, and participants were removed if inconsistent or contradictory answers were provided on these questions. Data were also removed for participants who typed responses deemed incomprehensible to two research assistants or took shorter than 2 min or longer than an hour to complete the study. Finally, the study was short in length (designed to take approximately 15 min) to help maintain engagement and attention.

##### Data analysis

All analyses were completed in the R statistical environment (version 4.4.1; R Core Team, 2024) using the psych (Revelle, 2025), lavaan (Rosseel, 2012), semTools (Jorgensen et al., 2025), furniture (Barrett & Brignone, 2017), and tidyverse (Wickham et al., 2019) packages. More detail regarding each step of the analytic process is provided below.

##### Descriptive statistics

In this initial step, we computed descriptive statistics about the PROCS as well as the nature of the conversations evaluated (e.g., topic, goal, setting, conversation partner) across the two samples. Additionally, data visualizations were produced to provide information regarding the distribution of total scores and the distribution of individual items on the PROCS.

##### Parallel analysis and exploratory factor analysis

Using the first participant sample, parallel analysis was conducted to estimate the number of factors in the construct, which is important in determining whether a single composite score is appropriate or whether multiple subscale scores are warranted. Simulated and resampled eigenvalues were compared between one and ten factors to determine the number of factors that balanced variance explained, the parsimony of the model, and random variation. Given the non-normal distributions of items, polychoric correlations were used for this analysis.

Exploratory factor analysis (EFA) was conducted to estimate item loadings, item uniqueness, variance, and correlations between items and between factors (if more than one factor). Although parallel analysis indicated one factor was sufficient, information gathered during previous phases of measure development highlighted the need to consider models with both one and two factors. Accordingly, models were conducted for both one and two factors. To adjust for the non-normal distribution of the items, polychoric correlation matrices and a robust diagonally weighted least squares estimator were used for the fitting of the models. All ten items were included in the EFA models with an oblique matrix rotation (i.e., “oblimin”) that allows factors to be correlated (for models with more than one factor). For the two-factor model, we calculated correlations between factors.

##### Confirmatory factor analysis

Using the second participant sample, confirmatory factor analysis (CFA) modeling was used to assess model structure, reliability, and validity of the measure. The main structure of the model was informed by the EFA results. As in the EFA models, to adjust for the non-normal distribution of the items, polychoric correlation matrices and a robust diagonally weighted least squares estimator were used to fit the models. Model-based fit statistics were computed for all models tested. These included root mean square error of approximation [RMSEA], comparative fit index [CFI], Tucker–Lewis index [TLI], standardized root mean squared residual [SRMR], and weighted root mean square residual [WRMR]. Model fit was evaluated using established criteria (Hu & Bentler, 1999; Schermelleh-Engel et al., 2003): RMSEA ≤ 0.06 (good fit), CFI ≥ 0.95 (good fit), TLI ≥ 0.95 (good fit), SRMR ≤ 0.08 (good fit), and WRMR ≤ 1.0 (good fit). For more information, see Bentler, 1972; Bentler, 2009; Chalmers, 2018; Cho, 2021; Cronbach, 1951; Fornell & Larcker, 1981; Green & Yang, 2009; McDonald, 199; Raykov, 2001; Zumbo et al., 2007; Zumbo & Kroc, 2019. Chi-square difference tests were conducted to compare nested models. Assumptions of the models were assessed for violations.

##### Reliability

Internal consistency (“reliability”) was evaluated using composite reliability (ω) and maximal reliability (coefficient H), which are more appropriate than Cronbach's alpha for scales that may violate tau-equivalence assumptions (McNeish, 2018; Revelle & Zinbarg, 2009). Reliability coefficients ≥ 0.90 were considered excellent, and ≥ 0.70 acceptable (Nunnally & Bernstein, 1994). These were estimated for both the one-factor and two-factor models to determine which solution provided a more reliable measurement.

##### Convergent and divergent validity

Convergent validity was assessed through correlations between PROCS scores and three criterion variables expected to correlate strongly with conversational success: (1) self-reported conversation success, (2) indication of whether the conversation was “good,” and (3) willingness to have a similar conversation again. Divergent validity was evaluated by examining relationships between PROCS scores and demographic variables (age, gender, race/ethnicity, geographic region) that were expected to show weak or non-significant correlations with conversational success perceptions.

##### Summed scores

To validate the use of summed scores for this measure, we tested whether items demonstrated essential tau-equivalence (equal factor loadings) by comparing a constrained model with equal loadings to an unconstrained congeneric model using chi-square difference tests.

#### Results

##### Descriptive statistics

Participants chose to evaluate conversations covering a broad range of topics, including work, family, religion, politics, vacations, past memories, and future plans. When asked to indicate the goal of the conversation, 73% of participants reported their conversation was primarily focused on solving a problem or sharing information. The other 27% of participants reported that the goal of their conversation was primarily to build a relationship, establish rapport, or get to know their conversational partner. The majority of the conversations occurred at the participants’ homes (64%). Other reported settings included work (9%), outside (8%), the conversation partner’s home (8%), and entertainment venues (2%). 84% of participants reported that the conversation they selected was with a close family member or friend. Twelve percent reported a conversation with an acquaintance, and 4% with a stranger.

**Fig. 4:**
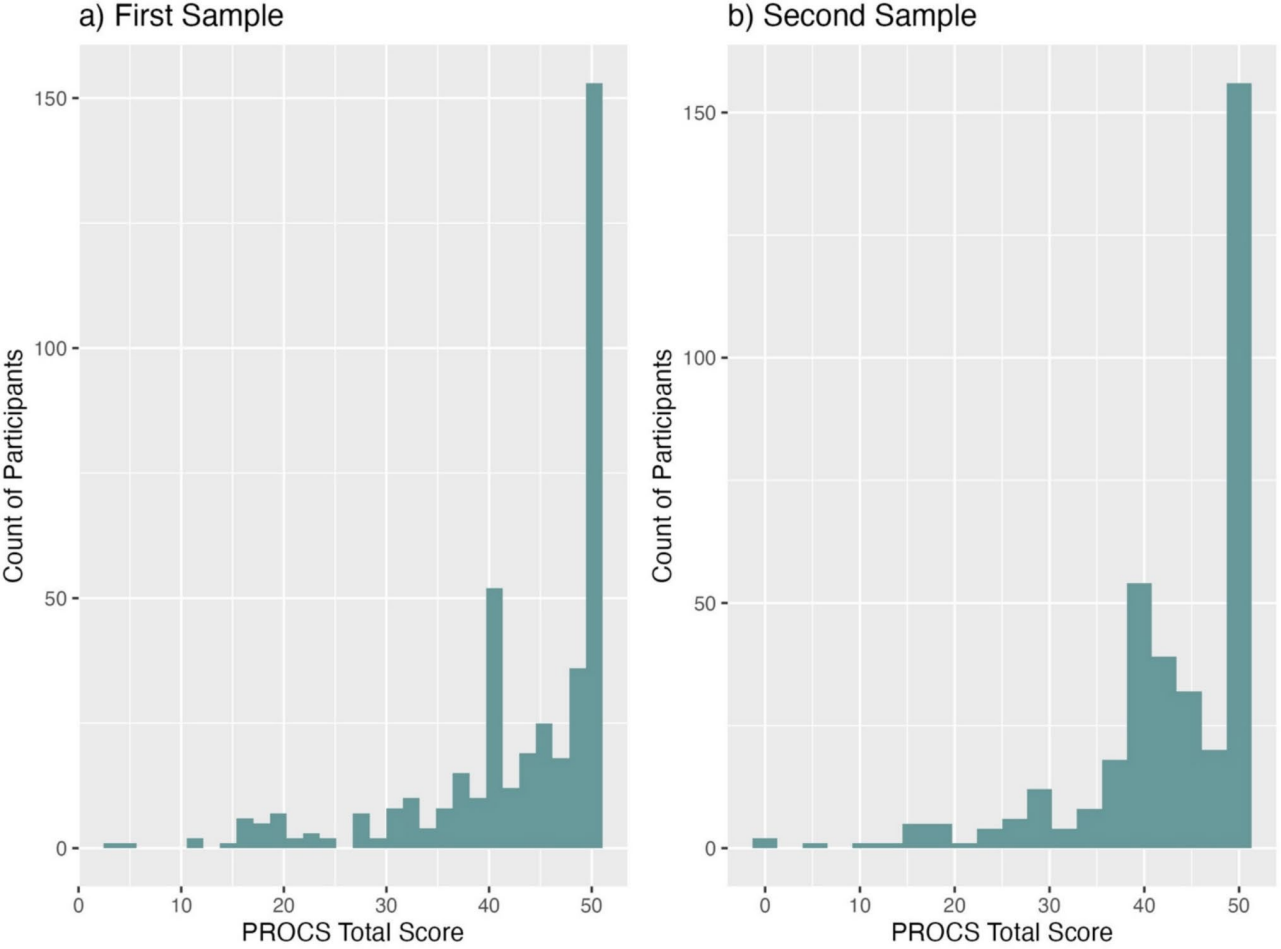
Distribution of total PROCS scores for phase four

**Fig. 5:**
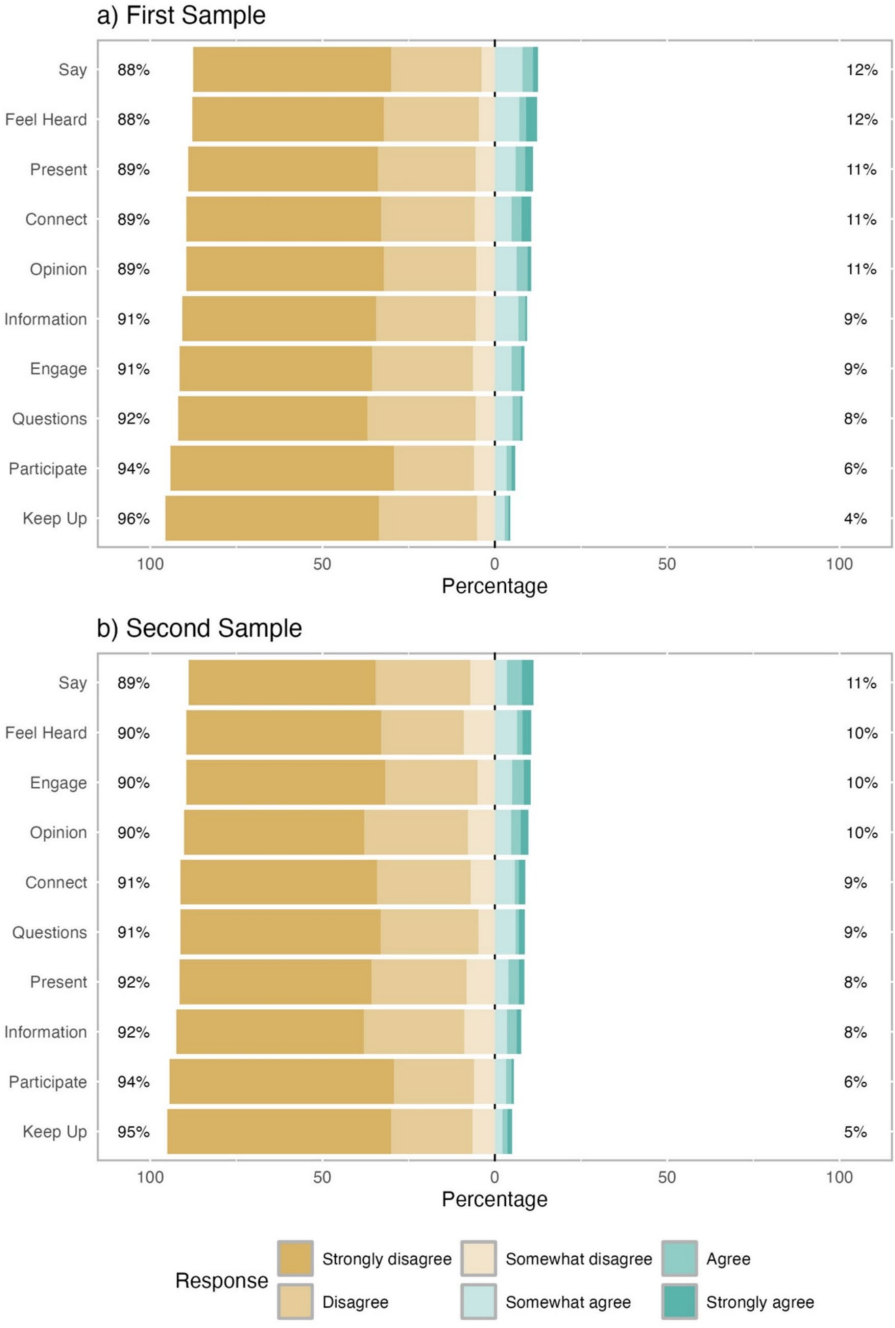
Distribution of each PROCS item for phase four

For the first sample, PROCS total scores ranged from 3 to 50 (*M* = 42.8, SD = 9.3, Mdn = 47.0) while the second ranged from 0 to 50 (*M* = 43.0, SD = 9.1, Mdn = 45.0). The distributions showed significant negative skew (skewness = – 1.62), suggesting that most conversations were rated positively (see Fig. 4). Individual item scores are presented in Fig. 5, highlighting the strong left skew across all items. The most highly skewed items (i.e., items with most positive responses) were “keep up” and “participate” while “say” and “feel heard” had the most negative responses.

##### Parallel analysis and exploratory factor analysis

**Fig. 6:**
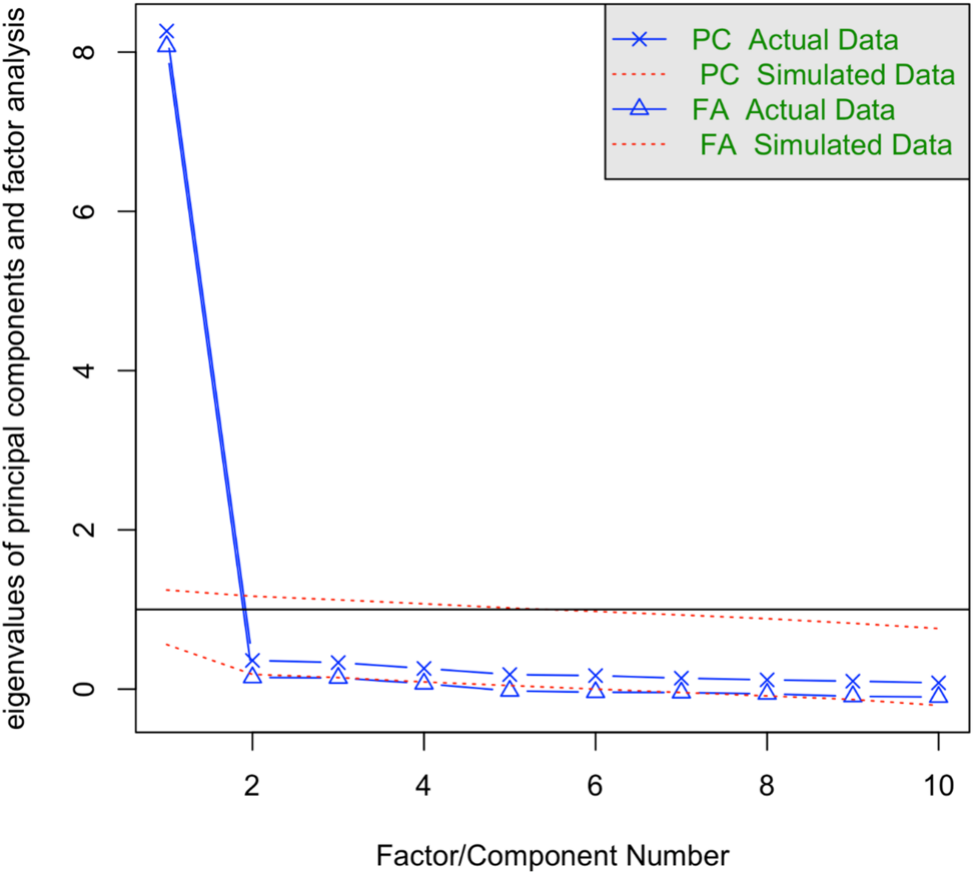
Results of the parallel analysis to determine number of factors in the PROCS construct

Parallel analysis was conducted using 1000 randomly generated datasets to determine the optimal number of factors. Results indicated that one factor was sufficient, as the eigenvalue for the first factor (λ = 8.26) exceeded both the 95th percentile (λ = 1.03) and mean (λ = 0.476) of randomly generated eigenvalues, while the second factor eigenvalue (λ = 0.146) fell below these thresholds (see Fig. 6). However, as previously mentioned, both one- and two-factor EFA models were tested.

Results for one- and two-factor EFA models are shown in Table 2. For the one-factor model, all loadings were statistically significant (*p* < 0.01) and substantial in magnitude (range 0.85–0.93), indicating strong relationships between items and the underlying factor. The two-factor model revealed that all items loaded primarily onto the first factor (loadings > 0.83), with minimal and largely non-significant loadings on the second factor (highest loading = 0.37, most < 0.10). The correlation between factors in the two-factor model was weak (*r* = 0.204), suggesting limited distinctiveness of the second factor.

**Table 2:** Exploratory factor analysis results of one- and two-factor models

	One factor			Two factors			
Keyword for candidate items	Loadings	Unique variance	Communalities	Factor 1 loadings	Factor 2 loadings	Unique variance	Communalities
Engage	0.867	0.249	0.751	0.863		0.248	0.752
Questions	0.908	0.175	0.825	0.911		0.174	0.826
Opinion	0.913	0.167	0.833	0.921		0.163	0.837
Information	0.930	0.135	0.865	0.953	– 0.097	0.12	0.88
Keep up	0.864	0.253	0.747	0.896	– 0.138	0.228	0.772
Feel heard	0.893	0.202	0.798	0.872	0.090	0.200	0.800
Present	0.913	0.166	0.834	0.832	0.371	0.044	0.956
Connect	0.921	0.152	0.848	0.875	0.171	0.144	0.856
Say	0.889	0.21	0.79	0.881		0.209	0.791
Participate	0.922	0.15	0.85	0.967	– 0.191	0.105	0.895
Sum of squared loadings	8.141			8.093	0.272		
Variance explained	0.814			0.809	0.027		

Blank loadings signify the loading is smaller than 0.05. All shown loadings are significantly different from zero at the 0.01 level

Item-level analysis revealed that all items contributed unique variance beyond the common factor, with commonalities ranging from 0.747 (“keep up”) to 0.865 (“information”). The proportion of unique variance was lowest for “participate” (0.15) and highest for “keep up” (0.253), indicating that while items share substantial common variance, each provides distinct information about conversational success and should be retained.

The one-factor model explained 80.7% of the total variance. Further, the average variance extracted for the one-factor model was 81.4%. Thus, the one-factor model that includes all items captures a high amount of the variation in the latent construct. Both factors together in the two-factor model explained 83.5% of the total variance,^4^ with Factor 2 accounting for less than 3% of the variance (compared to over 80% for Factor 1). Thus, while a multi-factor model almost always explains more variance than a one-factor model, the increase in this case is small.

While the two-factor model achieved slightly better fit indices, the improvement was minimal and not practically meaningful. The chi-square difference test indicated a non-significant difference (Δχ^2^[9] = 16.7, p = 0.053), suggesting that the single-factor model may fit just as well as the two-factor model. Most critically, the WRMR for the one-factor model (1.007) was only marginally above the acceptable threshold (1.0), while all other indices met or exceeded good fit criteria. Given the theoretical rationale, parsimony principle, and minimal practical improvement in fit, the one-factor model was selected as the preferred solution.

##### Confirmatory factor analysis

Using the information obtained in the EFA modeling (single factor, all ten items loading onto this factor), we confirmed the model structure and fit to the data using CFA modeling. The specification of the main CFA model allowed all items to load on the singular latent variable with all loadings, residuals, and variances to vary. The model estimates, fit statistics, and reliability estimates are presented in Table 3. Standardized loadings varied from 0.804 (for Keep Up) and 0.920 (for Connect).

**Table 3:** Loadings, fit statistics, and reliabilities of the main confirmatory factor analysis model

Item	Raw estimate	Standardized estimate
Engage	1.000	0.874
Questions	1.027	0.897
Opinion	1.041	0.909
Information	1.042	0.911
Keep Up	0.920	0.804
Feel Heard	1.007	0.880
Present	1.045	0.914
Connect	1.052	0.920
Say	1.042	0.911
Participate	0.993	0.868
Fit statistics		
CFI	0.9992	
TLI	0.9990	
RMSEA	0.0557	
SRMR	0.0279	
Reliability		
Omega	0.9592	
H	0.9704	

##### Reliability

This model demonstrated excellent internal consistency: composite reliability (ω = 0.959) and maximal reliability (H = 0.970). These values substantially exceed the threshold for excellent reliability (≥ 0.90; Nunnally & Bernstein, 1994) and approach the upper bound for psychological measures, indicating that PROCS items consistently measure the same underlying construct. The high reliability values indicate that PROCS scores demonstrate minimal measurement error and can be considered stable indicators of perceived conversational success. These reliability estimates support the measure's suitability for both research applications and clinical assessment contexts.

##### Convergent and divergent validity

Extending the main CFA model, we included correlations between the latent PROCS scores and three similar constructs: 1) self-rating of success, 2) indication of whether the conversation was good, and 3) willingness to have a similar conversation again, which were 0.573, 0.746, and 0.613, respectively. The correlation between PROCS scores and all three constructs represents a large effect size (Cohen, 1988), suggesting convergent validity exists for the PROCS measure.

The latent PROCS scores were not significantly correlated with age (*r* = – 0.003 *p* = 0.946), race (*r* = 0.134, *p* = 0.098), ethnicity (*r* = 0.129, *p* = 0.247), nor geographic location (*r* = 0.047, *p* = 0.408). It was significantly correlated with gender (*r* = 0.168, *p* = 0.011), with males rating conversations slightly higher than females; however, this represents a small effect size.

##### Summed score

To determine whether items equally contribute to the construct and validate the use of summed scores, we tested essential tau-equivalence by comparing two nested models: a tau-equivalent model with all factor loadings constrained to be equal versus a congeneric model with freely estimated loadings. Results showed a significant difference between the models (Δχ^2^[9] = 106.35, *p* < 0.001), indicating that the constraint of equal loadings significantly worsened model fit and at least one loading differs from the others. However, the variability in the standardized loadings is relatively small, ranging from 0.804 to 0.912 (see Table 3). Thus, given these small (and likely unmeaningful) differences, rejecting a simple model allowing each item to be equally weighted in a summed score is likely unwarranted (Widaman & Revelle, 2023).

## Discussion

Conversations are a fundamental part of human lives, and the outcomes of these interactions are key predictors of well-being and life satisfaction. While researchers have long sought to understand more about what makes communicative interactions successful, carefully developed and psychometrically validated tools are surprisingly lacking. Additionally, though clinical services for people with communication disorders should be focused on improving conversational outcomes, high-quality tools that can be used to track conversational success do not exist. Accordingly, this study developed and validated the PROCS, a tool for researchers and clinicians to quantify conversational success.

Creation of the PROCS included four crucial study phases. The first three phases focused on initial tool development and included iterative refinement based on researcher discussion, results from pilot testing, and user feedback. In the first phase, an initial draft of the PROCS was created through an in-depth review of established tools and existing literature, and extensive discussion among experts in the field. In the second phase, the PROCS was further refined through cognitive interviews with potential users. These included additional conversational experts (i.e., speech-language pathologists) and other individuals of different ages, genders, and life experiences. Importantly, both individuals with and without communication disorders were included in the cognitive interviewing process to ensure that the thoughts and ideas of people with a range of conversational experiences were included and to ensure the clinical relevance of the tool. By gaining input from a wide range of people, we were able to confirm that measure items were reflective of success (i.e., construct validity) and were unambiguous and easy to understand. This also ensured that all aspects of conversational success were captured within the measure (i.e., content validity) while allowing us to remove redundant or unnecessary items to keep the measure brief and parsimonious. In a third step, we assessed the utility of the PROCS in embodied, real-time conversations. As before, evaluation of the PROCS during this phase included participants with and without communication disorders. Further, it included an evaluation of different types of conversations to ensure its versatility across conversational settings. After this phase was completed, we made final decisions regarding the item bank content and tool structure based on user feedback and evaluation of measure outcomes.

The fourth and final phase focused on formal psychometric validation of the established version of the PROCS. Analysis suggested that the PROCS measures a unidimensional construct with each item demonstrating uniqueness while also relating strongly to the overall construct. This suggests that each item is important, and as such, all items were included in the final version of the tool. The finding of strong reliability suggests the measure is precise; convergent validity suggests the measure is correlated strongly to other measures of success; divergent validity shows it is unrelated to age, race, ethnicity, and geographic location—variables that success should not depend on. While tau-equivalence testing indicated at least one loading differed from others, qualitative assessment of the standardized loadings shows these differences are small and likely meaningless. Accordingly, using summed scores is likely adequate for this measure (Widaman & Revelle, 2023). That is, in practice, users of the PROCS can sum each item to obtain a total score that accurately represents the construct.

Of note, the data in both phases three and four were negatively skewed, indicating that conversations in these phases were viewed as more successful than unsuccessful. Within the given contexts of both phases, such results are unsurprising. In phase three, conversations were highly structured, low-stakes, and occurred in a friendly and supportive environment. In phase four, participants were free to select any recent conversation and may have been more likely to recall positive conversations and/or select conversations that were socially favorable. Of note, in phase two, scores were significantly lower when participants recalled negative conversations than positive conversations, indicating that the tool is sensitive to differences in success where they exist. Thus, we do not see the skewed results in phases three and four as inherently problematic or indicative of an inability to distinguish successful from unsuccessful conversations. However, for studies focused on conversational breakdowns or treatment effects, researchers may wish to ensure that conversational contexts are challenging enough to reduce potential skew and/or ceiling effects.

Taken together, the result of these four study phases yields a rigorously developed tool with strong evidence of robust psychometric properties. This tool is designed to evaluate both in-the-moment and recently recalled conversations. While this increases the feasibility and versatility of the measure, researchers should take appropriate steps to control for biases that may occur in retrospective recall contexts. The final version of the PROCS consists of ten items, used to evaluate a single conversation. Each item consists of the stem “*In this conversation, it was difficult for me…”* followed by a statement about the conversation which interlocutors rate on a six-point scale from “*strongly disagree*” to “*strongly agree.*” Each item is given a score from 0 (i.e., strongly agree) to 5 (i.e., strongly disagree) and an overall score is obtained by summing scores from each item. Thus, overall scores can range from 0 (maximally *unsuccessful* conversation) to 50 (maximally *successful* conversation). The entire measure can be completed in less than 10 min. Space is also given for respondents to indicate the conversational topic, setting, partner relationship, and goal. This was provided as a convenience to researchers and clinicians who would like to gather relevant information about the type of conversation being evaluated. The tool can be found in the appendix, and an automatically-scored electronic version of the tool can be found at https://human-interaction-lab.github.io/PROCS-Form/ .

### Application

The PROCS can be used in both research and clinical settings. In terms of research, the PROCS fills a gap in available and validated tools for understanding conversational success. For instance, the PROCS could be used to identify interactional behaviors that improve or hinder conversational outcomes. Of course, these behaviors will likely vary by the context and type of conversation (e.g., Dideriksen et al., 2022; Wynn et al., 2024). Thus, the ability to use this measure across different conversational contexts is a key strength, allowing a comparative understanding of conversations across different settings. This versatility also allows it to be used across different fields and areas of study. For instance, in family therapy research, it could be used to understand the strategies that best support conversational success in familial contexts. For research focused on clinical populations, the tool can be leveraged to identify the treatment approaches that foster the most promising conversational outcomes within a given population. Medical researchers may rely on it to identify the best strategies for medical providers to foster conversational success with their patients. Beyond the behaviors that drive conversational success, the PROCS may be beneficial in exploring the consequences of conversational success. For instance, this tool can be used to understand the role of conversational success on workplace satisfaction, educational performance, therapy outcomes, customer/patient approval, and business transactions. The PROCS can also be used to answer theoretical questions about conversational success, such as how much perceptions of success differ across conversational partners, the effect of external factors (e.g., background noise, distraction, cognitive effort) on success, or the degree to which success can be predicted by an interlocutor’s characteristics. Many more applications are possible.

In addition to being used for research, the PROCS can be used in clinical practice. While clinicians currently have access to measures of long-term communicative success (e.g., CPIB), the PROCS is the first rigorously developed tool that can be used to evaluate a client’s success within individual interactions. Such a tool is highly beneficial. For instance, this tool could be used as part of an initial therapy evaluation to help establish goals and could also be used as a progress monitoring tool (although future research will be needed to determine the measure’s responsiveness to change). Further, the PROCS could be used in therapy to identify the unique conversational behaviors that improve conversational outcomes for a specific dyad. There are many features of the PROCS that make it ideal for clinical practice. The measure is free, quick, simple to administer, and repeatable. The versatility of the measure also makes it well-suited for examining the conversational success of clients across contexts and partners. Further, by including the perspectives of individuals with communication disorders within the tool development process, we have ensured its clinical relevance and utility. Research could further strengthen the tool’s utility for specific clinical populations through additional psychometric validation with these groups.

### Limitations and future directions

While the PROCS has been carefully developed to ensure its validity and reliability, there are some ways in which future adaptations may enhance its usefulness for specific populations. For instance, while this tool was developed with input from individuals with a variety of communication disorders, it was not feasible to represent every type of communication disorder in the cognitive interviewing process. Thus, applicability and interpretation may vary across different populations, and the instrument may need to be adjusted for certain populations (e.g., altering item wording, changing stem phrasing). Additionally, psychometric validation focused specifically on neurotypical populations. While this validation decision was made to ensure a large amount of control, future work could validate this tool’s performance for relevant populations. Additionally, PROCS development and validation focused exclusively on adults. However, with some minor refinements, this tool may prove beneficial for younger populations as well. Accordingly, future work aimed at adjusting this tool’s usage for children and adolescents is warranted.

Next, while we examined basic relationships between contextual and demographic factors and PROCS scores, a more detailed analysis would be beneficial. For instance, while the correlation between gender and PROCS scores was small, measurement invariance testing would be useful to explore how gender may influence the application of PROCS. Further, using measurement invariance testing to examine other factors (e.g., partner familiarity, conversation type) can assess whether the measure functions differently across subpopulations (e.g., whether an item has a different meaning or a different influence on the total score in one subpopulation than another). Finally, the PROCS holds promise as a progress-monitoring tool and as a means of evaluating the efficacy of specific treatment approaches. However, the tool’s responsiveness to change has not yet been established. Accordingly, determining this information is an important next step before it can be used to track treatment-related progress.

## Conclusion

The purpose of this project was to rigorously develop and validate a measure of conversational success. This resulted in the PROCS, a tool that can be used to measure conversational success from the interlocutor’s perspective within individual interactions. These steps included initial tool draft development, cognitive interviewing, tool assessment in embodied conversation, and a large-scale psychometric validation. The PROCS affords researchers and clinicians with a simple and valid methodology for measuring conversational success.

## Supplementary Information

Below is the link to the electronic supplementary material.

Supplementary file1 (PDF 419 KB)

## Data Availability

The measure included in the Appendix and at https://human-interaction-lab.github.io/PROCS-Form/. Data and supplemental participant information available at https://osf.io/k8gxc/.

## Footnotes

1 One participant was both a person with a communication disorders and a speech-language pathologist.

2 The order of the scale was switched to “Very Difficult” to “Not Difficult at All” so as to be consistent with the other versions of the measure and remove the need for reverse scoring.

3 The decision to use a six-point scale is also in line of prior literature which suggests that between 5 and 7 Likert scale categories is optimal in maximizing variability, reliability, and ease and efficiency of use (Preston and Coleman, 2000).

4 Average variance extracted is not applicable to multi-factor models with cross-loadings and, therefore, could not be calculated for the two-factor model.
