# Supplementary material for: The Person-Reported Outcome of Conversational Success (PROCS): Tool development and psychometric validation
Source: Behav Res Methods. 2026 Jun 10;58(7):194. doi: 10.3758/s13428-026-03063-4 (PMC13253778; doi:10.3758/s13428-026-03063-4)
Supplement: Supplementary file 1 — Supplementary file1 (PDF 419 KB) [file 13428_2026_3063_MOESM1_ESM.pdf]

Appendix

The Person-Reported Outcome of Conversational Success (PROCS)

Name: ______________________ **Date:** ______________________

Topic: ______________________ Setting: ______________________

Partner: ______________________

What was the goal of this conversation? ______________________

	Strongly Disagree	Disagree	Somewhat Disagree	Somewhat Agree	Agree	Strongly Agree
1. In this conversation, it was difficult for me to... engage at a level I felt good about.	☐	☐	☐	☐	☐	☐
2. In this conversation, it was difficult for me to... ask questions as desired.	☐	☐	☐	☐	☐	☐
3.. In this conversation, it was difficult for me to... share my opinion as desired.	☐	☐	☐	☐	☐	☐
4. In this conversation, it was difficult for me to.. share information as desired.	☐	☐	☐	☐	☐	☐
5. In this conversation, it was difficult for me to... keep up with the conversation.	☐	☐	☐	☐	☐	☐
6. In this conversation, it was difficult for me to... feel heard by my partner.	☐	☐	☐	☐	☐	☐
7. In this conversation, it was difficult for me to... present myself the way I wanted to.	☐	☐	☐	☐	☐	☐
8. In this conversation, it was difficult for me to... connect with my partner as desired.	☐	☐	☐	☐	☐	☐
9. In this conversation, it was difficult for me to... say what I wanted to say.	☐	☐	☐	☐	☐	☐
10. In this conversation, it was difficult for me to... participate.	☐	☐	☐	☐	☐	☐

Number of responses per column:						
	x 5	x 4	x 3	x 2	x 1	x 0
Score by column:						
Raw score (sum of column scores):						/ 50

© Wynn, Budge, Baylor, Barrett, & Borrie

Note: This tool is also available online at https://human-interaction-lab.github.io/PROCS-Form/
